# Exosomes released by keratinocytes modulate melanocyte pigmentation

**DOI:** 10.1038/ncomms8506

**Published:** 2015-06-24

**Authors:** Alessandra Lo Cicero, Cédric Delevoye, Floriane Gilles-Marsens, Damarys Loew, Florent Dingli, Christelle Guéré, Nathalie André, Katell Vié, Guillaume van Niel, Graça Raposo

**Affiliations:** 1 Institut Curie, PSL Research University, UMR144, CNRS, F-75248 Paris, France; 2 Structure and Membrane Compartments, Centre National de la Recherche Scientifique, UMR144, Paris F-75248, France; 3 Cell and Tissue Imaging Facility, Infrastructures en Biologie Santé et Agronomie (IBiSA), Paris F-75248, France; 4 Institut Curie, Centre de Recherche, Laboratoire de Spectrométrie de Masse Protéomique, Paris F-75248, France; 5 Laboratoires Clarins—31 chaussée Jules César, Pontoise 95300, France

## Abstract

Cells secrete extracellular vesicles (EVs), exosomes and microvesicles, which transfer proteins, lipids and RNAs to regulate recipient cell functions. Skin pigmentation relies on a tight dialogue between keratinocytes and melanocytes in the epidermis. Here we report that exosomes secreted by keratinocytes enhance melanin synthesis by increasing both the expression and activity of melanosomal proteins. Furthermore, we show that the function of keratinocyte-derived exosomes is phototype-dependent and is modulated by ultraviolet B. In sum, this study uncovers an important physiological function for exosomes in human pigmentation and opens new avenues in our understanding of how pigmentation is regulated by intercellular communication in both healthy and diseased states.

## Abstract (web summary)

The activity of melanocytes determines skin pigmentation, and is regulated by a tight dialogue with keratinocytes. Here, the authors show that exosomes released by keratinocytes have a direct effect on melanocyte function, and exosome content is dependent on skin phototype and is modulated by ultraviolet B radiation.

## Graphical abstract

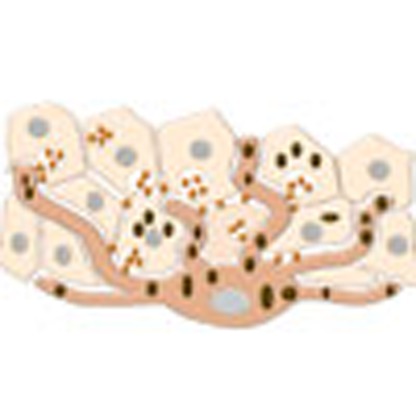

In the thin outermost layer of the skin, melanocytes and surrounding keratinocytes form the epidermal–melanin unit^1^. Solar irradiation activates signalling cascades that induce the secretion of molecules including hormones and growth factors that lead to increased melanin synthesis in melanocytes^2^,^3^,^4^,^5^. Skin pigmentation requires close intercellular communication and results in skin tanning but also constitutes an important defense mechanism for photoprotection against Ultraviolet B exposure. Cells communicate via either soluble, secreted factors or via membrane vesicles, commonly called extracellular vesicles (EVs)^6^,^7^. Exosomes are endosome-derived EVs and correspond to the intraluminal vesicles (ILVs) released into the extracellular environment on fusion of multivesicular bodies (MVBs) with the plasma membrane. Exosomes harbour membrane and cytosolic components such as proteins, lipids and RNAs^8^,^9^. In this study we show for the first time that, in addition to soluble factors^1^,^10^,^11^,^12^, normal human keratinocytes (NHK) release exosomes that play a role in the regulation of pigmentation. Exosomes carrying selected microRNAs (miRNAs) are targeted to melanocytes and modulate the pigmented status of melanocytes by altering gene expression and enzyme activity.

## Results

### MVBs polarize to intercellular contact sites

**Figure 1:**
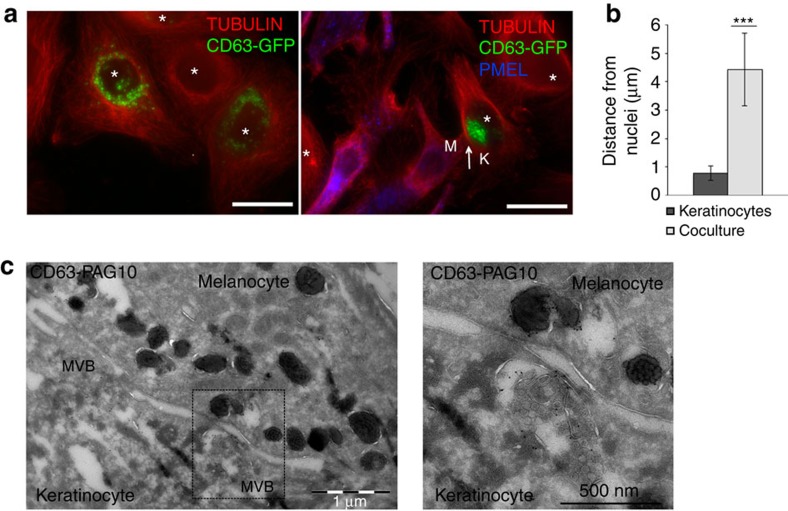
MVB polarization in cell culture and reconstructed epidermis. (**a**) CD63-GFP-transduced NHKs (green) in mono- or co-culture (ratio 1:1, melanocytes incubated with the same number of keratinocytes) with melanocytes were stained for tubulin (red) and PMEL (melanocyte-specific protein; blue) and were analysed using IFM (scale bar, 10 μm). Asterisks show the nuclei of keratinocytes and the arrow shows the site of contact between melanocyte and keratinocyte. (**b**) The distance of CD63-positive compartments from the centre of the corresponding nucleus was quantified in CD63-GFP-transduced NHK in mono- or co-culture with melanocytes (*n*=11; ***P<0.01, *t*-test). (**c**) EM analysis on ultrathin cryosections of Caucasian-reconstructed epidermis immunogold-labelled for endogenous CD63 (PAG 10 nm; scale bar, 1 μm). On the right, an inset corresponding to the magnified area of the back-boxed region depicts an MVB apposed to the keratinocyte plasma membrane in close association with melanocyte.

Previous studies reported that keratinocytes secrete vesicles with exosome-like features corresponding to the ILVs of MVBs^13^. Therefore, MVBs destined for secretion would be found in close proximity to the keratinocyte plasma membrane, as observed in other cell systems^14^. To visualize MVBs, NHKs were transduced with a lentivirus vector encoding CD63-GFP, a tetraspanin highly enriched in MVBs of most cell types^15^. After 3 days of transduction, immunofluorescence microscopy (IFM) showed that CD63-GFP-labelled compartments were primarily distributed around the nucleus (Fig. 1a, left panel). Interestingly, when transduced NHKs were co-cultured with normal human melanocytes a large fraction of CD63-positive compartments redistributed in NHK towards the areas of contact with melanocytes (Fig. 1a, right panel) as quantified by the increased distance of CD63-positive compartments from nuclei and relative to the control (Fig. 1b; *P*<0.01, *t*-test). Polarization of MVBs to the areas of cell–cell contact has similarly been observed in other exosome-secreting cell systems such as B and T cells^14^. Such a polarization is specific to MVBs since the distribution of early endosomes (EEA1) or Golgi (TGN46) was not drastically modified (Supplementary Fig. 1). To get further insight into such MVB redistribution in skin models, we analysed cell–cell contacts in the reconstructed skin epidermis (human epidermal model consisting of melanocytes and keratinocytes; see Methods) using immunoelectron microscopy (IEM). At high resolution, these observations revealed CD63-positive MVBs (labelling is for endogenous CD63) close to keratinocyte plasma membranes in the areas of contact with melanocytes (Fig. 1c), suggesting that those could potentially correspond to secretory MVBs.

### Keratinocytes secrete exosomes interacting with melanocytes

**Figure 2:**
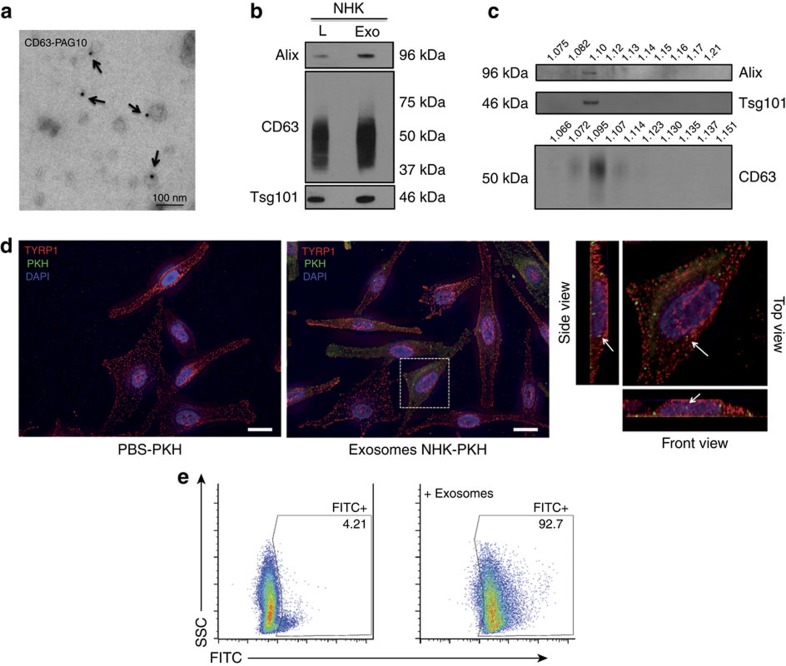
EV characterization and uptake. (**a**) EM analysis of exosomes from NHK immunogold-labelled for endogenous CD63 (PAG 10 nm, arrows). (**b**) WB analysis of exosomes (Exo; 10 μg) and total cell lysate (L; 20 μg) from NHK or fractions recovered after OptiPrep gradient (**c**) using anti-Alix, -CD63 and -Tsg101 antibodies. (**d**) Analysis by IFM of the interaction of PKH67-labelled (green) exosomes from NHK with melanocytes labelled for TYRP1 (red) and DAPI (blue; scale bar, 10 μm). (**e**) FACS analysis of melanocytes incubated for 1 h at 37 °C with FITC-labelled exosomes from NHK. Melanocytes incubated with the last wash of the FITC-labelling were used as a control.

We undertook a detailed characterization of EVs isolated from NHK medium with differential centrifugation. Supernatants contained 30- to 50-nm diameter vesicles partially labelled for CD63 as analysed using IEM (Fig. 2a and Supplementary Fig. 2a). The size of the vesicles observed using IEM is compatible with that reported for exosomes^9^ and western blot (WB) analysis revealed an enrichment for exosomal components in the EV fraction^16^ such as Alix, CD63 and Tsg101 when compared with whole-cell lysate (Fig. 2b). To control for the presence of potential contaminants, from cell organelles of non-endosomal origin or protein aggregates that can co-sediment during ultracentrifugation, the EV fraction was analysed by WB after isolation on an iodoxanol (OptiPrep) density gradient^17^. Alix- and Tsg101-positive vesicles secreted by keratinocytes were only present in the 1.1-g ml^−l^ fraction (Fig. 2c). CD63 was detected in three consecutive fractions (1.072–1.107 g ml^−1^; Fig. 2c) similar to what has been reported for exosomes released by other cell types^17^, indicating that the EV fraction consists mostly of a homogenous population of vesicles with features of exosomes. Reinforcing the exosomal nature of the vesicles secreted by NHK, the proteomic profile of the pellet after the last step of centrifugation revealed the presence of proteins that have been reported to be commonly present in exosomes from very different cell types, such as, for example, major histocompatibility complex class I, CD9, CD81 and Hsc70, but also cell-type-specific proteins such as keratins (Supplementary Data 1). Exosomes can transfer not only proteins but also miRNAs to target cells^18^,^19^. We analysed the miRNA profile of purified exosomes (Supplementary Data 2). Whereas most identified miRNAs have been previously reported to be expressed in NHK^20^, we also uncovered additional candidates (see below) that could be selectively sequestered within keratinocyte exosomes. Therefore, our collective results show that NHKs secrete EVs with features of endosome-derived exosomes, as shown by EM, WB, mass spectrometry and miR profiling^9^.

We hypothesized that keratinocyte exosomes are targeted to melanocytes to possibly modulate pigmentation. We tested the ability of NHK exosomes to interact with melanocytes by incubating these cells with PKH67-labelled exosomes (Fig. 2e). Exosomes not only interact with melanocytes but are internalized after 24 h of incubation as shown by Z projections of the imaged cells. We have also labelled exosomes with fluorescein isothiocyanate (FITC), and as analysed by fluorescence-activated cell sorting (FACS), a large majority of melanocytes (95±3%; mean±s.d. throughout) were FITC-labelled compared with the control (Fig. 2e), reinforcing the results that the NHK-isolated exosomes interact with melanocytes. In addition, NHK-derived CD63- green fluorescent protein (GFP)-positive exosomes, corresponding to a homogeneous population of vesicles (Supplementary Fig. 2c), were incubated with melanocytes. They were observed as punctate structures in contact with melanocytes (Supplementary Fig. 2d). Consequently, our data indicate that keratinocytes secrete exosomes that interact and are taken up by melanocytes.

### Exosomes regulate melanin synthesis by melanocytes

**Figure 3:**
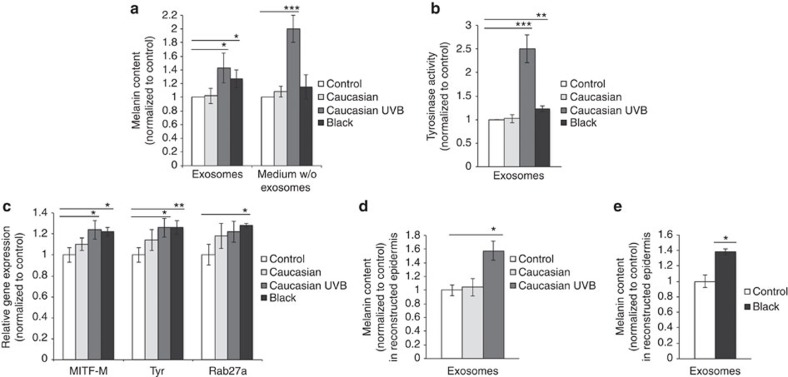
Exosomal effects on the regulation of pigmentation. (**a**) Analysis of melanin content (optical density at 492 nm) in Caucasian melanocytes incubated for 96 h with NHK exosomes (corresponding to 15 μg of protein) resuspended in PBS (left) and medium depleted of NHK exosomes (right; Caucasian, Caucasian irradiated with ultraviolet B and Black; ratio 1:1, melanocytes incubated with exosomes or medium isolated from the same number of keratinocytes). Melanocytes incubated without exosomes were used as a control. (**b**,**c**) Tyrosinase activity (**b**) or relative gene expression of Mitf-M, Tyrosinase and Rab27a (**c**) were measured in Caucasian melanocytes incubated for 96 h with exosomes from NHK (Caucasian, Caucasian irradiated with ultraviolet B and Black; ratio 1:1, 15 μg of exosomes). Melanocytes incubated without exosomes were used as a control. Intracellular melanin content analysis of light phototype-reconstructed epidermis incubated with exosomes from Caucasian, ultraviolet B-irradiated Caucasian (**d**) or Black (**e**) NHK. Reconstructed epidermis cultured only with medium and PBS were used as controls. Experiments in **d**,**e** were performed with different epidermis of the same phototype using different melanocyte donors. The data are from three independent experiments. Values are mean±s.d. (*P<0.05; **P<0.02; ***P<0.01, *t*-test, *n*=3).

To investigate the functional effects of NHK exosomes, we isolated exosomes from Caucasian (low phototype) and Black (high phototype) NHK, which could have potential different effects on pigmentation as the distinct Fitzpatrick phototypes have a different capacity to modulate melanin production^21^; we then investigated their effect on pigmentation. The intracellular melanin content (measured as optical density (OD) at 492 nm) of Caucasian melanocytes incubated with NHK exosomes from the same donor or the corresponding medium depleted of exosomes (supernatant of the last step of exosome purification; Fig. 3a) was not significantly changed in either of the conditions as compared with the control. In the skin epidermis, solar ultraviolet radiation stimulates the production of melanin^22^, and we tested whether NHK exosomes released after the ultraviolet B treatment of cells had an impact on pigmentation. Melanocytes incubated with exosome-depleted medium from ultraviolet B-irradiated NHK, which still contain soluble factors, increased their melanin content (≈2-fold). These observations corroborate with previous reports^23^ showing that ultraviolet B induces the secretion of soluble factors that stimulate melanogenesis. However, it is important to note that melanocytes incubated with exosomes secreted by ultraviolet B-treated NHK showed an increased melanin content as compared with melanocytes incubated with exosomes from non ultraviolet B-treated cells (43±22% and 2±11%, respectively; *P*<0.05, *t*-test). These observations lead us to evaluate whether such increased effects could be due to the secretion of increased amounts of exosomes. However, this was not the case as ultraviolet B exposure of NHKs did not affect the number of MVBs (Supplementary Fig. 3a) or the level of total protein in the exosomal pellet (Supplementary Fig. 3b). One hypothesis is that ultraviolet B treatment altered the exosome composition instead, which may then be required for the modulation of pigmentation. To further test the functional effects of exosomes on melanocytes, we isolated exosomes from Black NHKs. Black NHK secreted an equivalent amount of exosome-associated proteins as compared with Caucasian NHKs (Supplementary Fig. 3c). As observed with ultraviolet B-stimulated exosomes, the incubation of exosomes released from Black NHK (Fig. 3a, right plot, second bar), but not the corresponding depleted medium (Fig. 3a, right plot, fourth bar), increased the melanin content of Caucasian melanocytes (27±13% as compared with normalized control; *P*<0.05, *t*-test). Very interestingly, this indicated that secreted exosomes from high phototype NHK might intrinsically and without UV stimulation bear components that modulate melanin production. As a control we evaluated whether exosomes from another cell type could induce effects similar to ultraviolet B-stimulated NHK and Black NHK-derived exosomes, which was not the case (Supplementary Fig. 3d). Of note, in our assays melanocytes were incubated with exosomes recovered from a similar amount of keratinocytes. Extrapolated to the skin this ratio is far below the ratio of melanocytes and keratinocytes present in the epidermis (ratio 1:36). Therefore, the effects of exosomes *in vivo* could be expected to be even higher.

In order to further investigate their functional properties, we analysed the ability of exosomes to modulate the activity of tyrosinase (TYR), the key enzyme in melanin biosynthesis^23^. When Caucasian melanocytes were incubated with NHK exosomes from the same donor, TYR enzymatic activity did not significantly increase as compared with cells incubated without exosomes (Fig. 3b, second bar). However, a significant increase in TYR activity (150±30%) was observed when Caucasian melanocytes were incubated with ultraviolet B-stimulated exosomes from the same donor (Fig. 3b, third bar; *P*<0.01, *t*-test), further supporting a model where ultraviolet B stimulates the ability of exosomes to increase melanocyte pigmentation. Similarly, a 23±6% increase in TYR activity was observed when Caucasian melanocytes were incubated with exosomes from Black NHK (Fig. 3b, fourth bar; *P*<0.02, *t*-test), suggesting the effects of black keratinocytes in the stimulation of pigmentation via exosomes.

Given that exosomes increase pigmentation and TYR activity, we set out to examine whether they also affect expression of selected pigmentation genes such as microphthalmia-associated transcription factor (MITF), a master transcriptional melanogenesis regulator^24^,^25^. When Caucasian melanocytes were incubated with NHK exosomes from the same donor, no significant increase (10±6%) in the expression of MITF-M (the melanocyte isoform) was detected (Fig. 3c) correlating in agreement with the observation that no increase in pigmentation was observed (Fig. 3a). Accordingly, the expression of MITF-dependent genes, such as TYR and Rab27a, the latter encoding a GTPase involved in the mobilization of melanosomes to the cell periphery necessary for their transfer to keratinocytes^26^, was also unchanged (Fig. 3c). However, a significant increase in the expression of MITF-M (24±9%; *P*<0.05, *t*-test), TYR (26±9%; *P*<0.05, *t*-test) and Rab27a (22±10%) was observed in melanocytes incubated with ultraviolet B-treated exosomes from the same donor. The same observations were made with exosomes from Black donors (MITF, +22±4% (*P*<0.05); TYR, +26±6% (*P*<0.02); Rab27a, +28±2% (*P*<0.01); *t*-test; Fig. 3c). Together, these results indicate on one hand that ultraviolet B irradiation likely induces modifications in the composition of exosomes from Caucasian NHK, which then acquire the ability to stimulate pigmentation. On the other hand, exosomes from Black NHK already contain components that stimulate the pigmented phenotype of Caucasian melanocytes.

To further probe the pro-melanogenic effects of exosomes in a more physiological context, human-reconstructed pigmented epidermis was incubated with exosomes isolated from the cell culture supernatants of NHK. Reinforcing our previous results with cultured melanocytes, a significant increase in melanin content of the epidermis was observed with exosomes isolated from cell culture supernatants of ultraviolet B-irradiated Caucasian NHK (57±13%; Fig. 3d; *P*<0.05, *t*-test) and Black NHK (38±4%; Fig. 3e; *P*<0.05, *t*-test) when compared with the control epidermis (4±12%).

### Exosomes carry miRNA that regulate pigmentation

**Figure 4:**
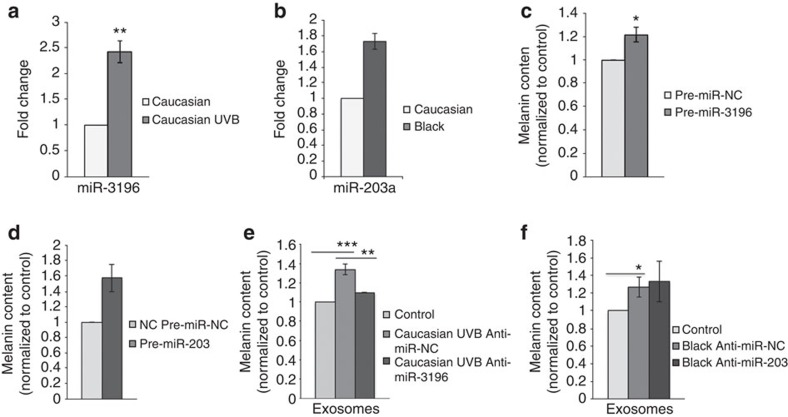
miRNA and pigmentation. (**a**) Fold change of miR-3196 in exosomes from Black NHK and normalized to exosomes from Caucasian NHK. (**b**) Fold change of miR-203-a in exosomes from Caucasian-irradiated NHK and normalized to exosomes from Caucasian NHK. (**c**–**f**) Analysis of intracellular melanin content in Caucasian melanocytes transfected with pre-miR-3196, (**c**) pre-miR-203 (**d**) or incubated for 96 h with exosomes from ultraviolet B-irradiated Caucasian NHK transfected with anti-miR-3196 (**e**) or with exosomes from Black NHK transfected with anti-miR-203. (**f**) Values are mean±s.d. (*P<0.05; **P<0.02; ***P<0.01, *t*-test, *n*=3).

The observations that miRNAs are possible modulators of pigmentation in melanocytes^27^,^28^,^29^ and that EVs sequester miRNAs^18^ led us to investigate whether the observed effects on melanin content and gene expression could be mediated by miRNAs contained within an exosomal vehicle as suggested above^30^. As a result, we analysed the miRNA composition of Black, Caucasian and irradiated Caucasian NHK exosomes (Supplementary Data 3). Of note, the miRNA profile of keratinocyte exosomes is modified after ultraviolet B exposure^31^. Interestingly, only one miRNA, hsa-miR-3196 for which a precise target has not been reported in the literature, was differentially expressed between irradiated and non-irradiated Caucasian NHK exosomes (Fig. 4a). In addition, more than 30 different miRNAs were differentially expressed between Caucasian and Black exosomes (Supplementary Table 1). Of note, miR-203 was highly expressed in Black exosomes (Fig. 4b), and this miR was recently reported to be a key regulator of melanogenesis in melanoma cells, increasing pigmentation and TYR protein levels^32^.

To directly evaluate the involvement of these miRNAs in pigmentation, Caucasian melanocytes were transfected with either pre-miR-3196 or pre-miR-203 (Supplementary Fig. 4a,b). Pre-miR-3196 transfection increased the intracellular melanin content of Caucasian normal human melanocyte (21.5±6.3% as compared with the control; Supplementary Fig. 5c; *P*<0.05, *t*-test) and the gene expression of MITF-M (395±98.7%; *P*<0.01, *t*-test) and of Rab27a (225±6.3%; *P*<0.01, *t*-test) without greatly modifying TYR expression (Supplementary Fig. 5a). Pre-miR-203 transfection increased the intracellular melanin content (57.5±17%; Fig. 5d; *P*<0.01, *t*-test) and, in contrast to mir-3196, enhanced the expression of TYR (41±6.8%; *P*<0.01, *t*-test) and Rab27a (17±4.3%; *P*<0.01, *t*-test; Supplementary Fig. 5b). MITF-M gene expression was not modified, suggesting that miR-203 may act on TYR regulation independently of the MITF pathway. Similarly, these results reinforce a role of miR-203 in the upregulation of TYR in primary melanocytes in addition to melanoma cells as reported^32^ and reveal for the first time a role for miR-3196 in melanogenesis. To further test the impact of these exosome-associated miRNAs on the process of pigmentation, Black and Caucasian ultraviolet B-irradiated keratinocytes were treated with anti-miR-203 or anti-miR-3196, respectively, to specifically downregulate their expressions and subsequent loading into exosomes (Supplementary Fig. 4c,d). Exosomes from Caucasian ultraviolet B-irradiated keratinocytes transfected with anti-miR-3196 lose the ability to upregulate melanin production in melanocytes (Fig. 4e). Exosomes from Black keratinocytes transfected with anti-miR-203 did not induce any significant variation as compared with exosomes from NHK transfected with anti-miR-NC (Fig. 4f), suggesting that additional exosome-associated components likely compensate for the lack of miR-203 or can even be expressed differently in these conditions. Additional molecules, such as proteins or lipids, might cooperate with the identified miRNAs and be partially responsible for the observed effects. In addition, we cannot rule out that such molecules might control other properties of exosomes, such as exosomes from Caucasian ultraviolet B-irradiated NHK pre-treated with trypsin, which failed to increase pigmentation (Supplementary Fig. 6). In sum, our results highlight that keratinocyte exosomes modulate their miRNA content to fine-tune melanocyte pigmentation via different signalling pathways (that is, miR-3196 and MITF-dependent or miR-203 and MITF-independent).

## Discussion

In this study we show for the first time that keratinocytes communicate with melanocytes via EVs with features of exosomes that carry miRNAs with the capacity to modulate pigmentation.

Keratinocytes have been previously reported to secrete exosomes containing stratifin that stimulate the activity of the metalloprotease MMP-1 in fibroblasts^13^. Our results further expand the characterization of the EVs secreted by keratinocytes, showing that the vesicles isolated from the cell culture supernatants of keratinocytes float on a density of 1.1 g ml^−l^ and express proteins present in the ILVs of MVBs (CD63, Tsg101 and Alix). The mass spectrometry analysis identifies proteins reported to be present in exosomes from different cell types^33^, also revealing the presence of stratifin as previously shown in keratinocyte exosomes^13^. The redistribution of CD63-positive MVBs towards the sites of contact with melanocytes is reminiscent of the observed polarization of MVBs that accompanies exosome secretion by immune cells^14^ as well as other cell types such as neurons and epithelium cells^34^. CD63-positive MVBs are observed in the human-reconstructed pigmented epidermis at the sites of contact with melanocytes, supporting the exosomal features of the EVs secreted by keratinocytes. Moreover, we have not observed vesicles larger than 110 nm in these pellets and we could not detect vesicles or other membranes in the pellet collected during differential ultracentrifugation (10,000*g*) indicating that keratinocytes secrete primarily small (30–100 nm) membrane vesicles. Although very unlikely, it cannot be ruled out that small plasma membrane-derived vesicles are also present in the vesicular fraction used in this study.

The EVs/exosomes released by keratinocytes once in contact with melanocytes induce increased TYR activity as well as expression of pigmentation genes that logically lead to the observed increase in melanin content of receiving melanocytes. Our data also highlight that exosomes secreted by keratinocytes from different phototypes and after ultraviolet B stimulation control melanocyte pigmentation to a different extent and likely by distinct mechanisms. Whereas exosomes secreted by Black keratinocytes have the ability to stimulate pigmentation to an even higher extent than the supernatant containing only soluble factors, exosomes secreted by Caucasian keratinocytes do not show any effect unless the keratinocytes are exposed to ultraviolet B at doses close to the physiological solar exposure. Although the supernatants depleted of exosomes induce melanin production, the exosomes by themselves also increase the pigmentation status of cells and reconstructed epidermis. The effects of exosomes are certainly due to their ability to interact with melanocytes and to be internalized as observed in other cell systems. Such an interaction will then allow delivery in the endocytic pathway of components that control the pigmentation process. We show that exosomes released by the different keratinocytes, Caucasian or Black, carry a distinct array of miRNAs. Our sequencing analysis reveals that miR-203 is overexpressed in exosomes secreted by Black keratinocytes. As previously reported, miR-203 targets Kif5b and regulates TYR expression and melanosome transport^32^, which may explain the observed effects of exosomes. Different from exosomes released by Black keratinocytes and on ultraviolet exposure, exosomes from Caucasian keratinocytes show an upregulation of miR-3196. Although this miR shows at least 50 targets as predicted by *in silico* analysis, our study now describes for the first time its involvement in the pigmentation process.

Collectively, our findings highlight a novel mode of communication between keratinocytes and melanocytes and attribute a novel function for exosomes in the regulation of skin pigmentation. This study also sheds new light on the understanding of how the pigmented phenotype is maintained and regulated. In addition to soluble factors released by keratinocytes^1^, exosomes, as membrane-enclosed vesicles carrying membrane proteins and cytosolic components, are likely to participate in the homeostasis of the skin. They could be involved in the modulation/maintenance of the pigmented status of the skin, a process that could be altered in disease. These studies open the path for new strategies to manipulate pigmentation in healthy and diseased states as several hyper- and hypopigmentation disorders affect individuals of all skin phototypes.

## Methods

### Antibodies and other reagents

Monoclonal antibodies and their sources were as follows: Rabbit polyclonal anti-β-tubulin (ab59680; 1:500), rabbit anti-EEA1 (ab2900; 1:200), horseradish peroxidase (HRP)-conjugated goat polyclonal antibodies to rabbit IgG (ab6721; 1:10,000) and to mouse IgG (ab6789; 1:10,000) were from Abcam. Mouse monoclonal anti-CD63 (CLB180; 1:200) was from Zymed (Invitrogen). Sheep anti-TGN46 (AHP500GT, 1:400) was from AbD Serotec. Mouse monoclonal anti-TSG101 (GTX70255; 1:500) was from GeneTex. Sheep anti-EGFR (20-ES04; 1:500) was from Fitzgerald. Rabbit anti-TYRP1 (sc-25543; 1:200) was from Santa Cruz Biotechnology. Anti-Alix (1:1,000) antibody was a kind gift from Pr. Remy Sadoul. Secondary goat anti-rabbit or anti-mouse antibodies conjugated to Alexa Fluor-488, -555 or -647 were from Invitrogen (1:200). Protein A-conjugated to 10-nm gold particles was from Cell Microscopy Center (AZU, Utrecht University, Utrecht, the Netherlands).

### Cell culture and ultraviolet B treatments

Human foreskin neonatal melanocytes and keratinocytes were obtained from Cellsystems. Keratinocytes were grown in DermaLife Basal Medium with DermaLife K Life Factors. Only keratinocytes from the second to fourth passage were used. Melanocytes were grown in DermaLife Basal Medium with DermaLife M Life Factors. Only melanocytes from the second to third passage were used. Keratinocytes were seeded and irradiated 24 h later with one shot of 30 mJ cm^−2^ of ultraviolet B (312 nm) using a Biosun machine (Vilber Lourmat, Suarlée, Belgium). Before irradiation, the medium was replaced with PBS, then immediately removed and replaced by the culture medium. Cell viability was determined by the trypan blue (Invitrogen) exclusion test. Keratinocytes were seeded in six-well plates, incubated overnight and ultraviolet B- or non-irradiated cells were counted after 24, 48 and 72 h.

### Lentiviral transduction of CD63-GFP

Keratinocytes were transduced with the lentiviral vector Cyto-Tracer pCT-CD63-GFP (System Biosciences). After 24 h the cells were washed with PBS and fresh medium was added to them. Conditioned medium was recovered after 48 h to isolate exosomes.

### miRNA transfection

Melanocytes were transfected with pre-miR-203, pre-miR-3196 or pre-miR-NC (as control) using Oligofectamine (Invitrogen). Cells were transfected a second time after 48 h and analysed after 96 h from the first shot of transfection. Keratinocytes were transfected with anti-miR-203, anti-miR-3196 or anti-miR-NC (as control) and conditioned media were recovered after 48 h to isolate exosomes.

### Reconstructed epidermis

Reconstructed pigmented epidermis (phototype IV, Sterlab, France) was incubated with growth medium (Sterlab) supplemented with total purified keratinocyte-derived exosomes for 2 days or PBS as a control. Then, the medium was replaced daily for 3 days. The epidermis were removed from their inserts and incubated for 1 h at 100 °C in 400 μl of SOLVABLE (Perkin Elmer) to digest the tissue. Melanin was quantitated on half of the sample by reading its OD at 490 nm. Results were normalized to the control, averaged and they represent a mean and s.d. of three independent samples.

### Exosome isolation

Exosomes were prepared from conditioned media for 48 h on subconfluent cells. Media were centrifuged at 2,000*g* for 15 min (4 °C) and 4,000*g* (15 min, 4 °C) to remove debris. Then, the supernatant was centrifuged at 10,000*g* for 30 min (4 °C) and the exosomes were collected from the supernatant by centrifugation at 100,000*g* for 60 min (4 °C), rotor Ti45. The supernatant after the last step of 100,000*g* (depleted media) was used to test the effects of soluble factors. The pellet was resuspended and washed in PBS, pH 7.5 as described^35^. The amount of proteins was quantified and 1.5 × 10^6^ keratinocytes release ∼15 μg of exosomes.

For the OptiPrep density gradient, a discontinuous iodixanol gradient was prepared, 40, 20 and 10% from a stock solution (Sigma) with 0.25 M sucrose, 10 mM Tris pH 8 and 1 mM EDTA. The sample was overlaid on the bottom of the gradient and centrifugation was performed at 350,000*g* for 60 min at 4 °C, rotor SW41. Ten fractions of 490 μl each were collected. The fractions were diluted with PBS, centrifuged at 100,000*g* for 60 min, resuspended in 30 μl of PBS and stocked at −20 °C.

To value the role of proteins, exosomes were treated with 0.25% trypsin (Sigma) for 20 min at 37 °C and trypsin was then inhibited (Soybean trypsin inhibitor, Sigma) and washed with PBS.

### WB analysis

Cells were lysed on ice in lysis buffer (20 mM Tris, 150 mM NaCl, 0.1% Triton X-100, 1 mM EDTA, pH 7.2) with a protease inhibitor cocktail (Roche). Lysates or exosomes were incubated in a sample buffer with or without 350 mM 2-mercaptoethanol (Sigma), boiled for 5 min and fractionated with SDS–PAGE using Nupage (4–12%) Bis-Tris gels (Invitrogen) and transferred to nitrocellulose membranes (Millipore). The membranes were blocked in PBS/Tween 0.1% (PBS/T) with 5% non-fat dried milk, incubated with the indicated primary antibody diluted in PBS/T, washed four times in blocking solution and incubated with HRP-conjugated secondary antibody followed by washing in PBS/T. Blots were developed using the ECL Plus Western blotting detection system (GE Healthcare) according to the manufacturer's instruction.

### Proteomics

*In-gel digestion*: Proteins were migrated on a SDS–PAGE gel (10%). Briefly, following SDS–PAGE and washing of the excised gel slices, proteins were reduced with 10 mM dithiothreitol before alkylation with 55 mM iodoacetamide. After washing and shrinking the gel pieces with 100% acetonitrile, in-gel digestion was performed using trypsin (Sequencing Grade, Promega) overnight in 25 mM ammonium bicarbonate at 30 °C.

*Liquid chromatography–MS/MS analysis*: The extracted peptides were analysed with nano-LC-MS/MS using an Ultimate3000 system (Dionex S.A.) coupled with an LTQ-Orbitrap mass spectrometer (Thermo Fisher Scientific, Bremen, Germany). Samples were loaded on a C18 precolumn (300 μm inner diameter × 5 mm; Dionex) at 20 μl min^−1^ in 5% acetonitrile, 0.1% TFA. After 3 min of desalting, the precolumn was switched online with the analytical C18 column (75 μm inner diameter × 15 cm; C18 PepMapTM, Dionex) equilibrated in 95% solvent A and 5% solvent B (5% acetonitrile, 0.1% formic acid and 80% acetonitrile, 0.085% formic acid). Bound peptides were eluted using a 5–52% gradient of solvent B for 57 min at a 200-μl min^−1^ flow rate. Data-dependent acquisition was performed on the LTQ-Orbitrap mass spectrometer in the positive ion mode. Survey MS scans were acquired in the Orbitrap on the 475-1,200 *m/z* range with the resolution set to a value of 60.000. Each scan was recalibrated in real time by co-injecting an internal standard from ambient air into the C-trap (‘lock mass option'). The five most intense ions per survey scan were selected for CID fragmentation and the resulting fragments were analysed in the linear trap (LTQ). Target ions already selected for MS/MS were dynamically excluded for 180 s.

*Processing of MS data*: Data were acquired using the Xcalibur software (version 2.0.7) and the resulting spectra were then analysed via the MascotTM Software created with Proteome Discoverer (version 1.4, Thermo Scientific) using the SwissProt Homo sapiens (human) Protein Database. Carbamidomethylation of cysteines, oxidation of methionine and protein N-terminal acetylation were set as variable modifications for all Mascot searches. Specificity of trypsin digestion was set and two missed cleavage sites were allowed. The mass tolerances in MS and MS/MS were set to 2 p.p.m. and 0.8 Da, respectively. The estimated false discovery rate of all peptide and protein identifications was less than 1%, by automatically filtering on peptide length, mass error and Mascot score of all peptide identifications.

### Interaction of exosomes with melanocytes

FITC-labelled exosomes were used to characterize the binding and/or the internalization of exosomes from keratinocytes to melanocytes. The Fluoreporter FITC protein labelling kit (F-6434, Molecular Probes) was used to label exosomes, according to the manufacturer's instructions. Exosomes were labelled and the excess of FITC-reactive solution was removed by rinsing in 1 ml PBS. The final washing supernatant was used as the negative control in the flow cytometry analysis. Data were acquired on a BD Accuri C6 flow cytometer and analysed in FlowJo.

Purified exosomes resuspended in PBS were labelled with PKH67 (Sigma), as control PBS without exosomes was used. The uptake was performed at 37 °C for 24 h by incubating melanocytes with the same number of NHK from which exosomes were recovered. Cells were then fixed with 2% PFA (paraformaldehyde) and staining with anti-TYRP1.

### TYR activity assay

TYR activity was determined as previously described^36^ with slight modifications. Melanocytes were treated with exosomes from keratinocytes (ratio 1:1, melanocytes incubated with exosomes from the same number of keratinocytes) for 96 h and then suspended in phosphate buffer containing 1% Triton X-100. After vortexing, cells were clarified by centrifugation at 13,000 r.p.m. for 10 min at 4 °C. TYR (10 μl) substrate L-Dopa (15 mM) was incubated with 90 μl of extract in a 96-well plate for 30 min at 37 °C. The absorbance was read at 470 nm.

### Melanin assay

Melanocytes were treated with exosomes from keratinocytes or medium depleted of exosomes (ratio 1:1, melanocytes incubated with exosomes or medium isolated from the same number of keratinocytes) for 96 h and then disrupted by sonication in 50 mM Tris-HCl, pH 7.4, 2 mM EDTA, 150 mM NaCl, 1 mM dithiothreitol and protease inhibitors. Pigment was pelleted at 20,000*g* for 15 min at 4 °C, rinsed once in ethanol/ether (1:1) and dissolved in 2 M NaOH/20% dimethylsulfoxide at 60 °C. Melanin content was measured as OD at 492 nm (ref. 37).

### Quantitative real-time PCR

Total RNA was extracted from the control and treated cells or exosomes using the RNeasy Mini kit (Qiagen) or mirVana miRNA isolation kit (Ambion). The same amount of cDNA was synthesized using NCode VILOmiRNA cDNA synthesis kit (Invitrogen) and random primers. Real-time PCR was carried out with the LightCycler 480 (Roche), using the Syber green fluorescent probe, and normalized using the ribosomal gene S26 (for mRNA) or RNU6 (for miRNA). MITF-M: Fw 5′-TCTACCGTCTCTCACTGGATTGG-3′; Rw 5′-GCTTTACCTGCTGCCGTTGG-3′. TYR: Fw 5′- TGCCAAGCATCCTATCTTCC-3′; Rw 5′-CCATGTAGGATTCCCGGTTA-3′. S26: Fw 5′-CCGTGCCTCCAAGATGACAA-3′; Rw 5′-CGAATGACGAATTTCTTAATGGCCT-3′. Primers for Rab27a, miR-203, miR-3196 and RNU6 were from QIAGEN.

### Immunofluorescence microscopy

Cells were grown on coverslips at 70% confluency and then rinsed in PBS and fixed for 15 min in 4% paraformaldehyde/PBS at room temperature. Fixed cells were washed in PBS and quenched for 10 min in PBS/50 mM glycine, saturated in PBS containing 1 mg ml^−l^ BSA (blocking buffer) and permeabilized in PBS/0.05% saponin/1 mg ml^−1^ BSA (incubation buffer, IB). Cells were incubated for 1 h with the primary antibody diluted in IB, washed three times in IB and incubated with the corresponding secondary antibodies diluted in IB for 45 min. Coverslips were washed three times with IB and then mounted in DABCO medium and examined on an Eclipse 80i Upright Microscope (Nikon) equipped with a CoolSNAP HQ2 CCD Camera, a Piezo Flexure Objective Scanner and × 100 Plan Apo objective (1.4 numerical aperture CFI (chrome-free infinity)). Images are maximum-intensity z projections of three-dimensional image stacks acquired every 0.2 μm using the Metamorph software (MDS Analytical Technologies, Sunnyvale, CA).

### Electron microscopy

For ultrathin cryosectioning and immunogold labelling, the cells were fixed with 2% PFA or with a mixture of 2% PFA and 0.2% glutaraldehyde in 0.1 M phosphate buffer, pH 7.4. Cells were processed for ultracryomicrotomy (70 nm of thickness), immunolabelled with anti-CD63 and immunogold-labelled using PAG10 as reported^38^. For EM of the isolated exosomes, a drop of exosomes suspended in PBS was deposited on Formvar-carbon-coated electron microscopy grids, fixed as above, immunolabelled and stained using the method described for ultrathin cryosections^38^. All samples were analysed using a FEI CM120 electron microscope (FEI Company), and digital acquisitions were made with a numeric camera (Keen View; Soft Imaging System, SIS, Germany).

### Image analysis and quantification

Quantification of exosome size and number, and MVB number was determined using the iTEM software (Soft Imaging System).

### miRNA array profiling

All experiments were conducted at Exiqon Services, Denmark. The quality of the total RNA was verified by an Agilent 2100 Bioanalyzer profile. Total RNA (140 ng) from sample and reference was labelled with Hy3 and Hy5 fluorescent label, respectively, using the miRCURY LNA microRNA Hi-Power Labeling Kit, Hy3/Hy5 (Exiqon Services) following the procedure described by the manufacturer. The Hy3-labelled samples and a Hy5-labelled reference RNA sample were mixed pair wise and hybridized to the miRCURY LNA microRNA Array 7th (Exiqon Services), which contains capture probes targeting all miRNAs for human, mouse or rat registered in the miRBASE 18.0. The hybridization was performed according to the miRCURY LNA microRNA Array Instruction manual using a Tecan HS4800 hybridization station (Tecan, Austria). After hybridization the microarray slides were scanned and stored in an ozone-free environment (ozone level below 2.0 p.p.b.) in order to prevent potential bleaching of the fluorescent dyes. The miRCURY LNA microRNA Array slides were scanned using the Agilent G2565BA Microarray Scanner System (Agilent Technologies Inc., USA) and the image analysis was carried out using the ImaGene 9.0 software (BioDiscovery Inc., USA). The quantified signals were background-corrected (Normexp with offset value 10, and normalized using the quantile normalization method, which we have found produces the best between-slide normalization to minimize the intensity-dependent differences between the samples.

### Data availability

The proteomics data set can be downloaded from http://xfer.curie.fr/get/nil/quPNEagSx9J/raw.rar

## Additional information

**How to cite this article:** Lo Cicero, A. *et al.* Exosomes released by keratinocytes modulate melanocyte pigmentation. *Nat. Commun.* 6:7506 doi: 10.1038/ncomms8506 (2015).

## Supplementary Material

Supplementary Information Supplementary Figures 1-6, Supplementary Table 1.

Supplementary Data 1 List of exosome-associated proteins from NHK identified by Liquid Chromatography-MS/MS Analysis.

Supplementary Data 2 List of miRNAs identified in exosomes from NHK.

Supplementary Data 3 Changes in miRNAs expression in exosomes from Caucasian (NHK C 1, 2, 3), UVB irradiated Caucasian (NHK C UV 1, 2, 3) or Black (NHK B 1, 2, 3) NHK (each condition done in triplicate).
