# Supplementary material for: Exosomes released by keratinocytes modulate melanocyte pigmentation
Source: Nat Commun. 2015 Jun 24;6:7506. doi: 10.1038/ncomms8506 (PMC4491833; doi:10.1038/ncomms8506)
Supplement: Supplementary Information — Supplementary Figures 1-6, Supplementary Table 1. [file ncomms8506-s1.pdf]

Supplementary materials:

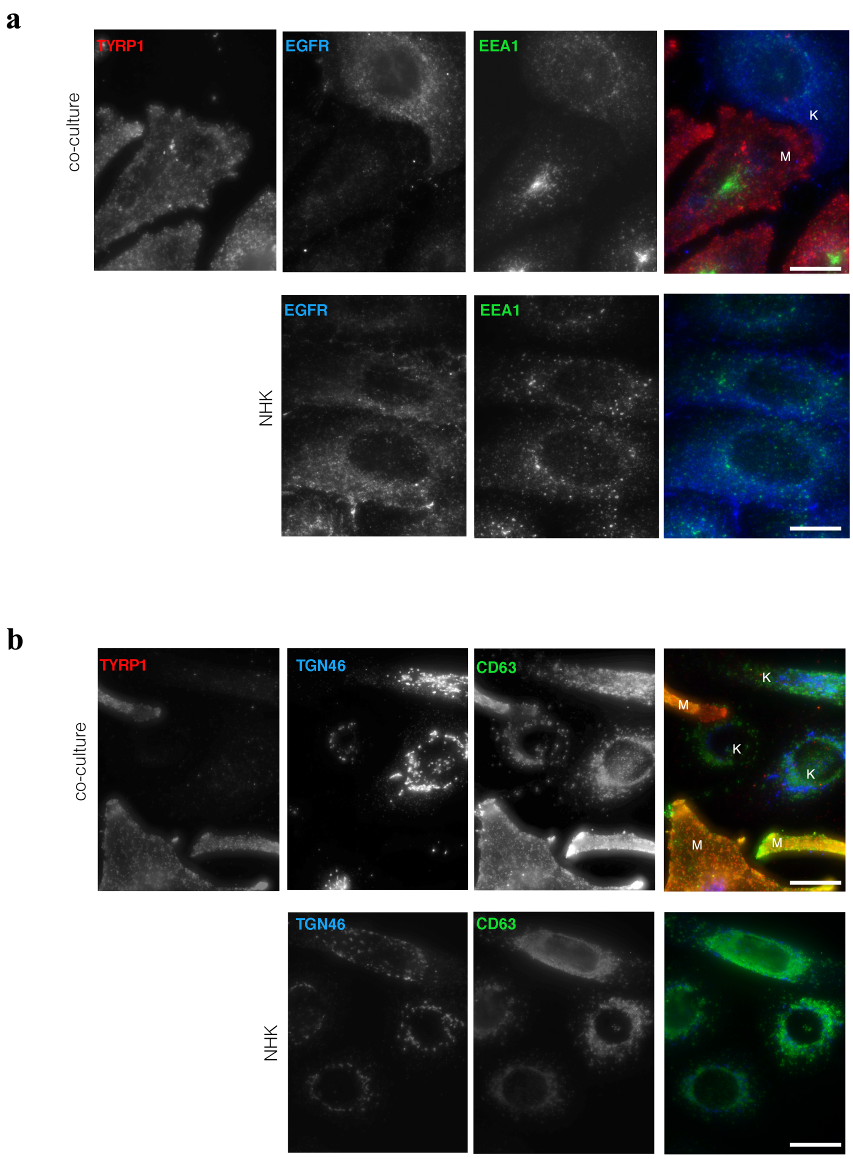

Supplementary figure 1

Supplementary Figure 1

(a-b) IFM analysis of NHK in mono- or co-culture with melanocytes (24 h) labeled for EGFR (keratinocyte marker, blue), TYRP-1 (melanocyte marker, red) and EEA1 (early endosomes, green) (a) or TYRP-1 (red), TGN46 (Trans Golgi Network, blue) and CD63 (MVB, green)(b).

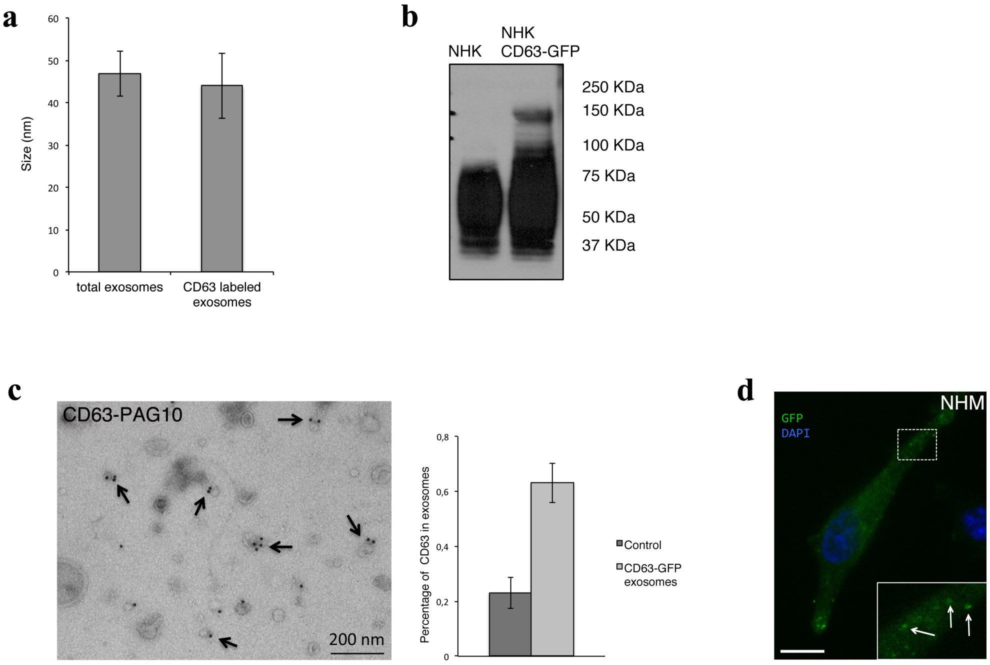

Supplementary figure 2

Supplementary Figure 2

(a) Average size of the diameter in nm of total exosomes population or CD63-labeled exosomes from NHK. (b) Western blot analysis of control NHK and CD63-GFP-transduced NHK (NHK CD63-GFP) cell lysates using anti-CD63 antibody. (c) EM analysis of NHK CD63-GFP exosomes immunogold-labeled for CD63 (PAG 10 nm). Graph indicates the percentage of CD63 in exosomes (gold particles) with (Control) or without CD63-GFP transduction (Number of exosomes determined using ITEM software). (d) Melanocytes (NHM) incubated 24 h with exosomes (white arrows) from NHK CD63-GFP were labeled for GFP (green) and DAPI (blue) and observed by IFM (scale bar: 10 μm).

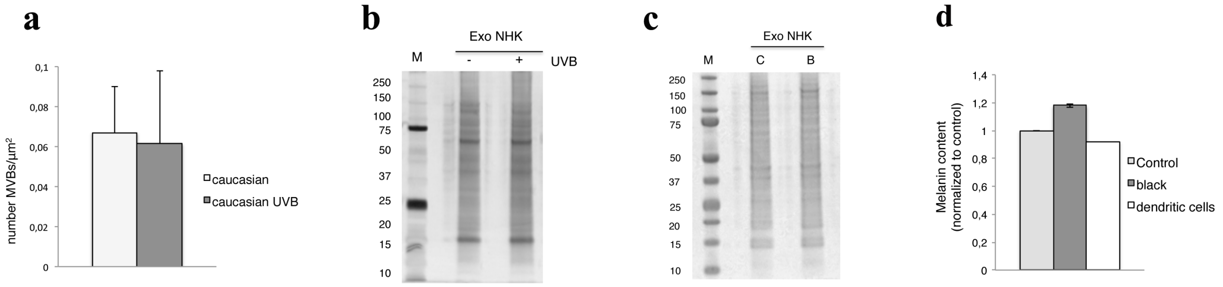

Supplementary figure 3

Supplementary Figure 3

(a) Quantification on ultrathin epon sections of the number of MVBs per surface (µm^2) in control or UVB-irradiated NHK (Number of MVBs was determined using ITEM software). (b) Comassie blue staining of exosomes from non- (-) or UVB-irradiated (+) Caucasian NHK. (c) Comassie blue gel staining of exosomes from Caucasian (c) or Black (b) NHK. Molecular weight (M) is in kDaltons. (d) Analysis of melanin content in Caucasian melanocytes incubated for 96 h with medium with PBS (Control), exosomes from Black keratinocytes and exosomes from dendritic cells.

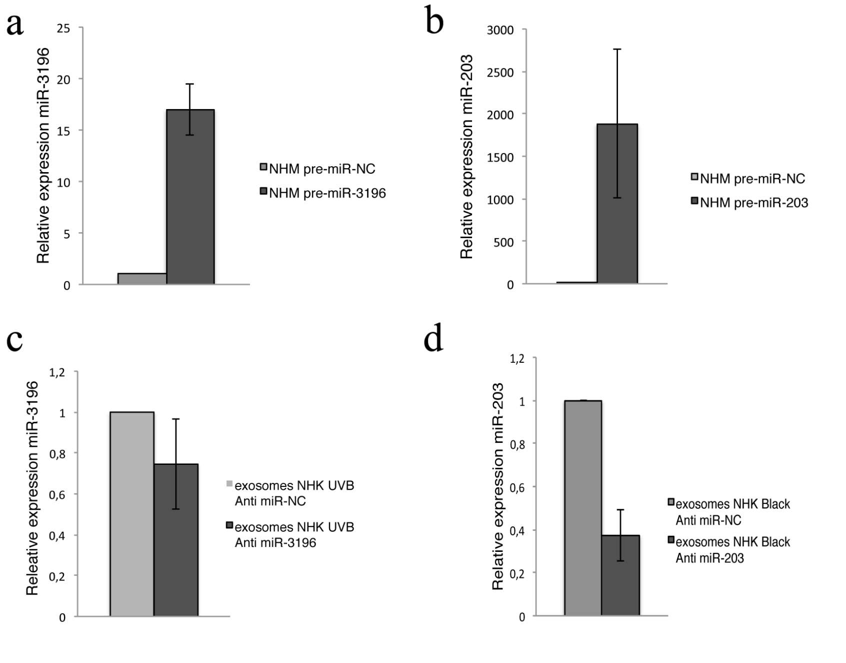

Supplementary figure 4

Supplementary Figure 4

Relative expression of miR-3196 and miR-203. Quantification of miR in melanocytes after transfection with pre-miR-3196 (a) or pre-mirR-203 (b). Quantification of miR in exosomes secreted by keratinocytes transfected with anti-miR-3196 (c) and anti-miR-203 (d).

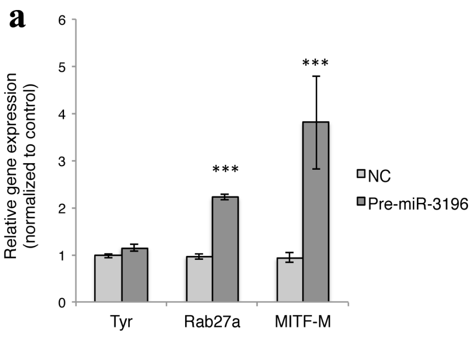

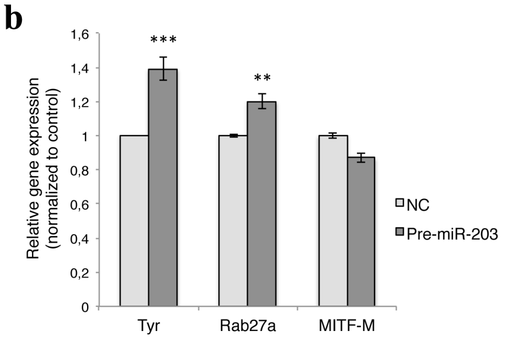

Supplementary figure 5

Supplementary Figure 5

Relative gene expression of Tyrosinase (Tyr), Rab27a and MITF-M in Caucasian melanocytes transfected with pre-miR-3196 (a), pre-miR-203 (b). Values are mean ± SD (*P < 0.05; **P < 0.02; ***P < 0.01)

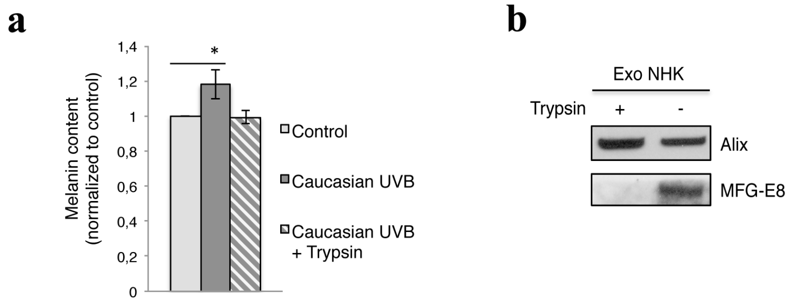

Supplementary figure 6

Supplementary Figure 6

(a) Analysis of intracellular melanin content in Caucasian melanocytes incubated with exosomes (96 h) from UVB-irradiated Caucasian NHK treated (gray stripes) or not (dark grey) with Trypsin (0,025%, 20 min at 37°C). (b) Western blot analysis of NHK exosomes (Exo) treated (+) or not (-) with Trypsin as in A using anti-Alix or -MFG-E8 (protein associated to exosome membrane) antibodies as a positive control for trypsinization efficiency.

.

Probe ID	Annotation	Average Hy3	logFC	p-value
42708	hsa-miR-99a-5p	6,022	1,134	4,18E-03
42923	hsa-miR-30c-5p	5,98	0,955	5,23E-03
32884	hsa-miR-342-3p	5,501	0,933	5,23E-03
10937	hsa-miR-132-3p	5,497	0,849	1,06E-02
168819	hsa-miR-200a-3p	5,325	0,867	1,06E-02
11004	hsa-miR-203a	5,559	0,796	1,09E-02
4610	hsa-miR-126-3p	6,49	0,651	1,09E-02
147880	hsa-miR-323b-3p	5,46	0,594	1,09E-02
169402	hsa-miR-625-3p	5,752	0,597	1,09E-02
42739	hsa-miR-339-5p	5,521	1,032	1,09E-02
46438	hsa-let-7g-5p	6,538	0,606	1,18E-02
10985	hsa-miR-191-5p	5,521	0,726	1,35E-02
168668	hsa-miR-4732-3p	6,815	0,667	1,60E-02
42571	hsa-miR-129-1-3p	5,798	0,737	1,60E-02
169385	hsa-miR-4500	5,91	0,682	1,60E-02
13177	hsa-miR-143-3p	6,249	0,909	1,65E-02
169307	hsa-miR-4685-3p	6,117	0,885	1,65E-02
11041	hsa-miR-29c-3p	6,529	0,772	1,65E-02
146112	hsa-miR-30b-5p	6,178	0,859	1,69E-02
168769	hsa-miR-5002-5p	5,998	0,772	1,84E-02
146008	hsa-miR-26b-5p	6,982	0,456	1,87E-02
148228	hsa-miR-3656	8,229	-0,547	1,87E-02
29490	hsa-miR-7-5p	9,001	0,544	1,87E-02
147701	hsa-miR-491-3p	11,394	0,468	2,25E-02
42696	hsa-miR-943	7,967	-0,424	2,31E-02
29872	hsa-miR-340-5p	5,762	0,579	2,39E-02
168951	hsa-miR-548as-3p	5,896	0,944	2,43E-02
11040	hsa-miR-29b-3p	7,262	0,418	2,64E-02
42502	hsa-miR-204-3p	10,643	0,498	2,64E-02
148000	hsa-miR-3195	5,727	0,73	2,64E-02

Supplementary Table 1

miRNAs expression analysis for exosomes from Black NHK vs. exosomes from Caucasian NHK. The table shows differentially selected and expressed miRNAs candidates (i.e. microRNAs where the p-value is less than 0.05), ranked according to the p-value.
